# Results of a patient-oriented second opinion program in Germany shows a high discrepancy between initial therapy recommendation and second opinion

**DOI:** 10.1186/s12913-020-5060-7

**Published:** 2020-03-20

**Authors:** Jan Weyerstraß, Barbara Prediger, Edmund Neugebauer, Dawid Pieper

**Affiliations:** 1 University of Witten/Herdecke, Alfred-Herrhausen-Straße 50, Witten, 58455 Germany; 2 Institute for Research in Operative Medicine (IFOM), Interim Head: Prof. Dr. Rolf Lefering, Chair of Surgical Research, Faculty of Health, School of Medicine, University of Witten/Herdecke, Ostmerheimer Str. 200, 51109 Cologne, Germany; 3 Brandenburg Medical School Theodor Fontane (MHB), Brandenburg, Germany

**Keywords:** Second opinion, Second opinion program, Agreement, HRQoL

## Abstract

**Background:**

As of 2015, second opinions are legally implemented in Germany. However, empirical results from German second opinion programs are lacking. The aim of this study was to examine several aspects within a population of a German second opinion program.

**Methods:**

Study population consisted of patients who sought a second opinion in the period from August 2011 to December 2016. Multivariate logistic regression and ANOVA were used to examine differences in patient characteristics, differentiated by agreement of initial therapy recommendation and second opinion. Follow-up points for patient satisfaction and HRQoL were defined at 1, 3 and 6 months after obtaining the second opinion.

**Results:**

Total number of patients who sought a second opinion was 1414. Most common indications concerned the knee (37.3%), spine (27.3%), hip (11.5%) and shoulder (10.1%). The independent specialists did not confirm the initial therapy recommendations in two out of three cases. The type of indication influenced the agreement between initial therapy recommendation and the second opinion significantly (*p* = 0.035). The second opinion and the offered service was highly valued by the patients (89%).

**Conclusions:**

The second opinion offers patients the possibility to confirm a medical indication independently and support patients in their decision making process. Reasons for the large discrepancy between initial therapy recommendation and second opinion should be addressed in future research.

## Background

The term second opinion is defined as an independent assessment of a medical condition performed by a physician or specialist in addition to an initial medical diagnosis [1]. It was introduced in the USA for the first time in the beginning of the 1970’s when several sickness funds started to require an independent second opinion for particular surgical operations due to regional differences and excessive surgery rates [2–4].

In the last decades second opinions became available for other medical indications besides the elective surgery and patients were able to seek an additional independent opinion on voluntary basis [5]. Seeking a second opinion can help improving the diagnosis and treatment of medical indications and possibly preventing the patient of unnecessary operations [6, 7]. Especially for medical indications as cancer or operations of the spine or hip, consulting another expertise can help to clarify the diagnosis and/or needed therapy. As the choice of therapy can be difficult for a patient, it is important that the patients are supported in a way that allows more involvement in their decision making process [8]. The second opinion informs the patients about their medical indication in a way that they can weigh the need and the consequences of a therapy to consider a treatment as adequate for them. Existing literature about the benefit of second opinion providers can be considered as outdated and knowledge from Germany is still completely missing [9].

According to paragraph 27b of the fifth social code book, as of 2015 it is possible in Germany for any insurant of a statutory health insurance to seek an independent second opinion for planned surgery. So far, for around 90% of the German population insured statutory by one of the existing 110 sickness funds in Germany [10], only the three medical indications fortonsillectomy, tonsillotomy and hysterectomy apply as independent second opinion, for which the costs can be borne by the sickness funds, as declared in the adopted guideline by the Federal Joint Committee [11]. Furthermore, it is strictly regimented that the specialists sought for a second opinion are not allowed to have any benefits in financial terms of the patient. Nevertheless, most sickness funds offer their insurants the possibility to seek an independent second opinion on their own initiative. Beside the three named above, the sickness funds bear the additional costs for a second opinion on other surgical procedures, whereby they differ remarkably in the scope of procedures in which a second opinion can be sought [12]. There is a widespread offer of second opinion services in Germany. Medexo is one of the first national second opinion services, which cooperates with several sickness funds and provides an independent and comprehensible specialist opinion accessible for every patient.

The main purposes of this study were to identify the characteristics of patients, who are seeking a second opinion with the help of a German national second opinion service since 2011, to compare the initial therapy recommendation with the second opinion issued by the independent specialist, to investigate the satisfaction of the patients with the second opinion and the service and the patients’ health-related quality of life (HRQoL).

## Methods

### Study population

The population consisted of patients from the German second-opinion service Medexo GmbH (www.medexo.com), who sought a second opinion in the period of August 2011 to December 2016. Present study data was anonymized by Medexo before transmitting to the researchers. Criteria for inclusion were the presence of patient information on age, sex, medical indication and the initial therapy recommendation. Additionally, patients with data of 3-months follow-up regarding patient satisfaction and HRQoL were included. All study data derived from patients who gave their permission on using their data for scientific purposes.

### Setting and data collection

In the first instance, patients approached the second opinion service by recommendation of their insurance company or own initiative either via phone, a contact form or email. Costs for the second opinion were either borne by the insurance company or the patient paid for the second opinion themselves. After completing the questionnaire regarding general patient information, including questions on HRQoL as of 2016, and gathering all medical documents required for the concerned specialist later to make his/her assessment like pictures of Magnetic Resonance Imaging (MRI), Computed Tomography (CT) scan, X-ray or similar inquiries, all data was transmitted digitally or by post to Medexo. All medical data was reviewed for completeness by the medical team of Medexo and then redirected to one of the independent specialists, who are working in cooperation with Medexo. These specialist network consists of over 80 leading specialists with international recognition and many years of experience. Completeness of the medical information was necessary to guarantee an accurate second opinion and the patient was informed by Medexo if information is lacking. Once the specialist had revised the provided medical information in detail, he/she created his/her individual assessment of the patient’s medical indication - the second opinion. No direct contact between the independent specialist and the patient him−/herself took place at any time. Based on this assessment, a comprehensive second opinion in plain language was issued by the medical team of Medexo. Finally, Medexo provided the patient-initiated second opinion online on the user account to download or alternatively sent it per mail to the patient. It was possible for every patient to consult Medexo with questions concerning the second opinion at any time. Furthermore, patients could contact the independent specialist via the medical team of Medexo, if desired. For administrative purposes data on patient satisfaction with the second opinion and the provided service, the chosen therapy was collected from the patients in intervals of 1, 3 and 6 months. Since 2016 the SF-12® Health Survey was added to the inquiry to assess the patient’s HRQoL [13].

### Statistical analysis

Data used in this study concerned only orthopedic indications as this category represent more than 90% of all medical indications.

For the overview of the population, descriptives were used and presented as mean and the standard deviation (SD) for all continuous variables and percentages for all categorical variables. Continuous variables were the patient’s age at registration, time to receive the second opinion and the SF-12® Physical (PSC-12) and Mental (MSC-12) Component Summary Scales. Categorical variables were the patient’s sex, agreement between initial therapy recommendation and second opinion and the medical indication. The variable “agreement between initial therapy recommendation and second opinion” was defined as the agreement between initial therapy recommendation and second opinion for the overall therapy category, e.g. physiotherapy or knee surgery. If these two therapy recommendations differed from each other, a “no” was given for the variable. Multiple imputation was applied in 133 of 1414 cases for the primary outcome variable “agreement between initial therapy recommendation and second opinion” due to missing values. It was performed by creating a new big dataset consisting of the original dataset with 133 missings for the 1414 cases plus the 10 new imputed datasets. In each of the imputed datasets the 133 missings were replaced by randomly imputed values based on the non-missing data for this variable. This step was necessary to ensure unbiased estimates and a higher validity compared to complete case analysis [14]. In the next step all patient’s characteristics were analyzed by the agreement between initial therapy recommendation and second opinion with a multivariate logistic regression, while correcting for the influence of each separate patient characteristic. Patient satisfaction was analyzed separately by age groups and sex with a multivariate logistic regression for binary variables and a Two-way ANOVA for categorical variables, while correcting for the influence of each separate variable. For the evaluation of the SF-12® Health Survey answers, the corresponding syntax was used to obtain the patient’s SF-12® Physical (PSC-12) and Mental (MSC-12) Component Summary Scales ranging from 0 to 100 with 0 as the worst and 100 as the best score [13]. The statistical tool to perform the analyses was IBM SPSS statistics 22 (International Business Machines Corporation, NY, USA). Significance level was set at 5%.

### Reporting guideline

The guideline used for reporting was the Strengthening the Reporting of Observational Studies in Epidemiology (STROBE) statement where appropriate [15].

## Results

**Fig. 1:**
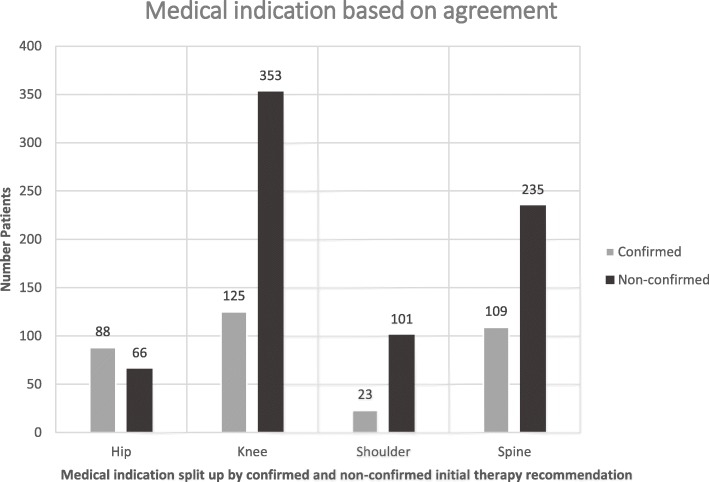
Distribution of the four most common medical indications by confirmed and non-confirmed initial therapy recommendation

In Table 1 the characteristics of the population are presented. Since the start in 2011 the number of patients seeking a second opinion has increased from 51 to 413 until 2016 (Total number of patients = 1414). On average, patients received their second opinion after 5 days. Most common medical indications concerned the knee (*N* = 524, 37.3%), the spine (*N* = 384, 27.3%), the hip (*N* = 161, 11.5%) and the shoulder (*N* = 142, 10.1%). About two-thirds initial therapy recommendations were not confirmed by the independent specialists. When patient’s characteristics were analyzed by the agreement between initial therapy recommendation and second opinion, only medical indication was found to be statistically significant with a *p*-value of .035. Figure 1 shows the distribution of the four most common medical indications hip, knee, shoulder and spine by confirmed and non-confirmed initial therapy recommendation. The greatest discrepancies were present for indications concerning the shoulder, the knee and the spine with approximately 81.5% (*n* = 101), 73.8% (*n* = 353) and 68.3% (*n* = 235) non-confirmed initial recommendations, while for the other medical indications the non-confirmation rate was below 50%. Furthermore, no other characteristics could be identified which differed significantly in between the patients. Patient’s satisfaction about the second opinion and the provided service is shown in Table 2. Around 89% (*N* = 335) and 84% of the patients (*N* = 255) were satisfied or very satisfied with the second opinion and their choice of therapy respectively. Moreover, 83% (*N* = 182) of the patients perceived the result of the second opinion as clearly or very clearly stated and 93% (*N* = 208) evaluated the text as understandable or very understandable. Around 60% (*N* = 152) of the patients chose the therapy recommended by the second opinion and 64% (*N* = 232) indicated that the second opinion was supportive or very supportive in their choice of therapy. We found no significant effect for the association of age and sex with the satisfaction of the patients with the second opinion and the provided service.

**Table 1:** Baseline characteristics of the study population

	Number patients (*N* = 1414)	Mean ± SD/%
**Sex:**	1197	100.0%
Male	649	54.2%
Female	548	45.8%
Missing	217	
**Age at reg. (Years):**	1183	58,2 ± 15.1
Missing	231	
**Agreement initial therapy recommendation and second opinion:**	1281	100,0%
Confirmed	451	35,2%
Non-confirmed	830	64,8%
Missing	133	
**Medical indication:**	1414	100,0%
Cardiology	30	2,1%
Feet	53	3,8%
Hand	33	2,3%
Hip	161	11,5%
Knee	524	37,3%
Shoulder	142	10,1%
Spine	384	27,3%
Other	87	5,6%
Missing	0	
**Time to receive second opinion (Days):**	251	5,1 ± 4,3
Missing	1163	

N = Total number of patients; SD = Standard deviation; Missing = Data missing at random due to changes in the data acquisition process.

**Table 2:** Patient’s satisfaction with the second opinion and the provided service

	Number patients (%)
**Are you satisfied with your second opinion?**	
Very satisfied	166 (44.0%)
Satisfied	169 (44.8%)
Unsatisfied	30 (8.0%)
Very unsatisfied	12 (3.2%)
**Which therapy have you chosen in the end?**	
Recommended therapy by second opinion	132 (59.7%)
Any other therapy	89 (40.3%)
**Are you satisfied with your choice of therapy?**	
Yes	255 (84.4%)
No	47 (15.6%)
**Would you consider the result of your chosen therapy as anticipated?**	
Yes	217 (76.1%)
No	68 (23.9%)
**How has your health condition changed 3 months after receiving the second opinion?**	
Distinct improvement	126 (34.2%)
Slight improvement	97 (26.3%)
No change	100 (27.1%)
Slight impairment	27 (7.3%)
Distinct impairment	19 (5.1%)
**Have you sought an additional medical opinion regarding your clinical picture next to the initial and second opinion?**	
Yes	152 (40.4%)
No	224 (59.6%)
**Did the second opinion support your choice of therapy?**	
Very helpful	113 (31.1%)
Helpful	119 (32.8%)
Slightly helpful	65 (17.9%)
Not very helpful	34 (9.4%)
Not helpful at all	32 (8.8%)
**Did the second opinion improve your understanding of your health condition or health problem?**	
Yes	271 (71.7%)
No	66 (17.5%)
Neither nor	41 (10.8%)
**Have your questions been answered (adequately) by the second opinion?**	
Just right	316 (84.3%)
Insufficient	49 (13.0%)
Too comprehensive	10 (2.7%)
**Did you perceive the text as understandable?**	
Very understandable	105 (46.9%)
Understandable	103 (46.0%)
Slightly understandable	13 (5.8%)
Not very understandable	2 (0.9%)
Not understandable at all	1 (0.4%)
**Did you perceive the result of your second opinion as clear?**	
Very clear	69 (31.4%)
Clear	113 (51.4%)
Slightly clear	24 (10.9%)
Not very clear	8 (3.6%)
Not clear at all	6 (2.7%)

**Fig. 2:**
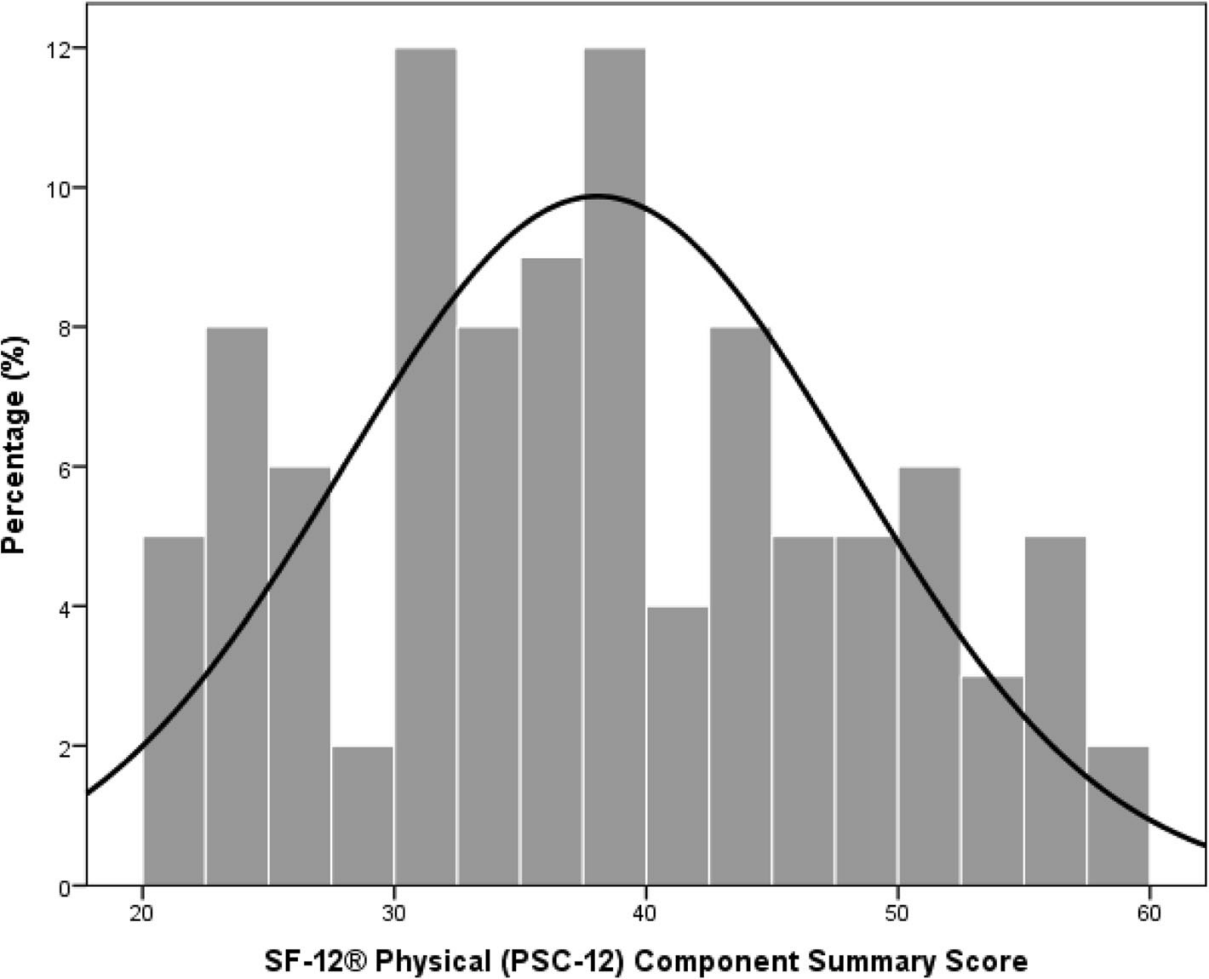
SF-12® Physical (PSC-12) Component Summary Scale of the patients

**Fig. 3:**
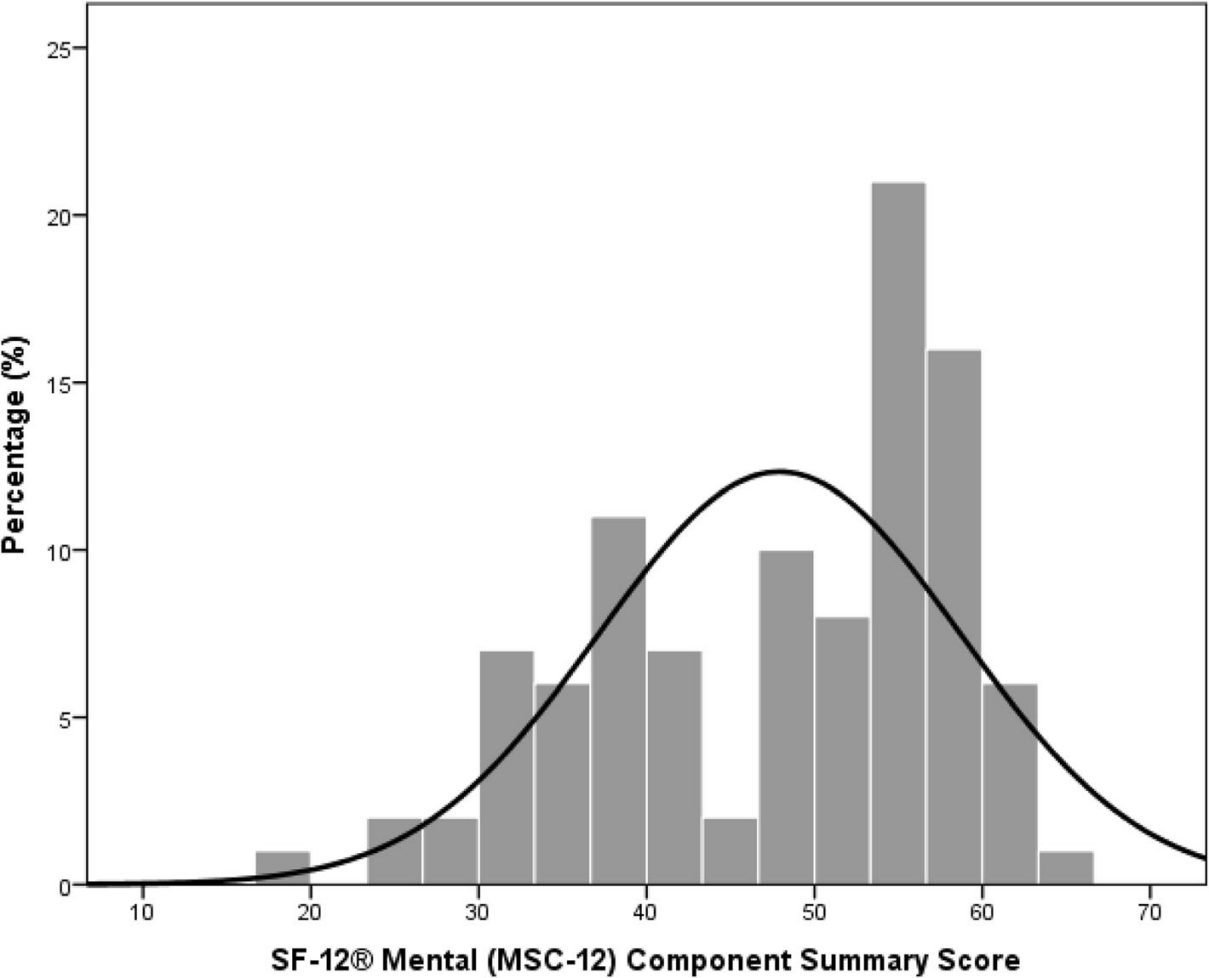
SF-12® Mental (MSC-12) Component Summary Scale of the patients

In Fig. 2 the SF-12® Physical (PSC-12) Component Summary Scale of the patients is presented. The mean PSC-12 summary score was 38 with a standard deviation of 10 for the physical health. The lowest PSC-12 summary score achieved was 20 and the highest 58. The SF-12® Mental (MSC-12) Component Summary Scale of the patients is shown in Fig. 3. The mean MSC-12 summary score was 48 with a standard deviation of 11 for mental health. The lowest MSC-12 summary score achieved was 17 and the highest 64.

## Discussion

Results of this article showed that only one third of the initial therapy recommendations were confirmed by the independent specialists underlying the importance of a possibility for patients to seek a second opinion. Patients sought a second opinion due to concerns with their knee, spine, hip and shoulder in about 88% of the cases. The type of medical indication was the only patient characteristic, which delivered significant discrepancies for the analysis on agreement between initial therapy recommendation and second opinion. Overall, the second opinion and the service of the second opinion service was considered as very satisfying by the patients and most patients chose the therapy recommended by the second opinion. Patient satisfaction with the second opinion and the provided service remained consistent as no significant discrepancies have been found for the age groups and sex.

Most studies assessing the second opinion are out of date and therefore only limited comparable [9]. However, substantial differences between the initial recommendation and the second opinion were also found in these studies. For instance, an agreement ranging from 58.6% up to 85.6% for medical indications concerning the knee was reported, while in this study the agreement for indications of the knee was only 26.2%. Varying agreements of 43–82% for several different medical indications have been found in a systematic review in 2015 with 13 included studies [16]. In another study, the results of the second opinion program “Best Doctors, Inc.” are reported [17]. “Best Doctors, Inc.” was the only study, which was highly comparable to Medexo regarding the patient acquisition and the independency of the patient-physician relationship. Results of this second opinion program showed that the second opinion led to a change in diagnosis or therapy in over 50% of the cases. These changes in diagnosis or therapy and therefore agreement varied noticeably per independent specialist.

Two-thirds non confirmed initial therapy recommendations and above 50% non confirmed initial diagnoses were also found by Lenza et al. in their recent study from 2017 including patients referred for spinal surgery [18]. Another study showed that the second opinion can be influenced if the specialist has knowledge of the initial therapy recommendation [19]. In this study orthopedic surgeons suggested an interventional therapy in more cases when knowing that the initial therapy recommendation was an intervention, compared to those orthopedic surgeons not knowing the initial therapy recommendation. These findings are also supported by the study of Philip et al. who found that about two-thirds of 65 oncologists interviewed rated the chance as high that the second opinion is influenced by the knowing of the initial therapy recommendation [20]. 41% of these oncologists also believed that the second opinion itself is of influential nature. A possible explanation for the differences in the level of agreement can be seen in the complexity of the medical indications with a remarkable number of treatment options and procedures to consider, e.g. surgical interventions versus conservative therapies [8]. This emphasizes, that advanced knowledge and expertise might be essential for the specialist to issue a second opinion.

In Germany, most sickness funds offer their insurants second opinion services.. It would be useful if these second opinion services would be scientifically evaluated to gain more knowledge about the benefit of these services in the Germany based on the different medical indications.

One satisfying finding in this study is the fact that patients received their second opinion on average after five days. This can be seen as one advantage of the independent second opinion compared to a second opinion issued by specialist with direct patient contact. The independent second opinion can be issued without any delay caused by full schedules of specialists, which is a common problem. Another advantage is that due to the broad offer of second providers an independent second opinion can be issued everywhere at any time. Therefore, patients with less possibility to seek a specialist in their region have no disadvantage due to their living situation any more.

Results from the survey on patient satisfaction show that with around 85%, the second opinion was perceived as satisfying and very satisfying. When compared to the literature, these findings can be considered consistent as Meyer et al. reported a satisfaction with the second opinion of 94,7% in their “Best Doctors, Inc” study [17]. In a German survey, 74% of the sample rated the opportunity to seek a second opinion as useful. The importance of the second opinion varied by severity of the health problem or recommended treatment [21]. In the same inquiry around 75% of patients, who already have experience with a second opinion, stated that the second opinion had led to a change in decision about a recommended therapy or treatment. The systematic literature review from Ruetters et al. in 2016 showed that patients are very satisfied with the second opinion and rated the second opinion as helpful and reassuring [16].

Patients tend to choose the therapy suggested by the second opinion as in this study almost 60% have indicated to have followed the recommendation of the second opinion, which is close to the reported 61,2% of patients who would follow the recommendation provided by the second opinion in the “Best Doctors, Inc.” study [17]. However, the satisfaction with the result of the chosen therapy did not differ for patients who followed the therapy recommendation of the second opinion and for patients who followed any other therapy recommendation. Any other therapy recommendation was chosen as comparison, because only a small percentage of patients followed an additional therapy recommendation next to the initial and second opinion. Moreover, the information was lacking if there was direct contact between the patient and the specialist, which means that only the second opinion had a guaranteed independency. Therefore, we wanted to discriminate the independent second opinion from a not independent or possibly not independent therapy recommendation. Reasons why patients seek a second opinion have been determined by Shmueli etc. al. in their cross-sectional national telephone survey in a sample of the Israeli population in 2017 [22]. Almost a third sought a second opinion for an orthopedic indication. 38% wanted to verify the initial therapy recommendation or had doubts about their treatment recommendation. Another interesting reason was the dissatisfaction with the first specialist also due to the fact that the patients didn’t feel informed enough about their condition and the therapy needed. Mellink et al. found that patients also sought a second opinion when receiving a diagnosis contradictory to what the patients believed they would get from the physician [23]. This includes also receiving a diagnosis of a serious health condition.

Next to the agreement and the satisfaction of the patients with the second opinion, the health status of the patients was analyzed with the Physical (PSC-12) and Mental (MSC-12) Component Summary Score. On average the patients scored 38 on the PCS-12 summary score and 48 on the MCS-12 summary scale with a standard deviation of 10 and 11 respectively. Comparing the results of the present study population to the German average score of 49 on the PCS-12 summary scale and 52 on the MCS-12 summary scale, patients only scored lower on the PCS-12 summary scale [24]. The lower PCS-12 summary score can be explained by the medical indications of the patients which were mainly of physical origin.

## Limitations

The present study is the first national study in Germany analyzing the second opinion in multilateral way. However, there were some limitations in this study. Study data was collected systematically by Medexo, mainly for administrative purposes. Due to changes in the data acquisition process, some patient characteristics had missing data at random. For some variables only information of a one or two year span was available. Data missing systematically could not be determined here. Furthermore, patients less satisfied with the second opinion could have tended to not answer the questions for the follow-up inquiry. Another limitation is the setting of the independent second opinion in this case. Although it has the advantage of independency, there is no direct contact between the patient and the specialist which means that the second opinion was issued by data review only without anamnesis. It is possible that assessments could differ if the specialist uses his self-gathered patient data instead of relying on other physician data. The main limitation can be seen in the study design. Next to that, it is questionable if patients seeking a second opinion are a representative sample of the general population. In the study of Shmueli et al. in 2017 certain characteristics for seeking a second opinion as female gender, living in central urban areas or serious health condition were identified [22]. The study population consisted only of patients who received a second opinion, a control group of patients without a second opinion is lacking. This problem was the case in earlier studies as well [9, 17] and needs to be scientifically evaluated in the future, taking into account the impact on the doctor-patient relationship [25]. Evaluation of the need of the second opinion with a study group of patients who received a second opinion and a control group with patients who did not receive a second opinion could be done by taking into account the perceived change of the patients’ health-related quality of life (HRQoL) since undergoing the recommended therapy.

## Conclusion

Medexo offers patients the possibility to obtain an additional independent medical opinion and thus an aid in decision-making. This offer is highly valued by patients and most patients have also followed the recommendation issued by the second opinion. More research on the second opinion with stronger study designs need to be done in the future. Hereby, reasons for the large discrepancy between initial therapy recommendation and second opinion can be investigated. In addition, surveys that can also map the economic dimension are desirable. Nonetheless, these results already show a very positive picture of the second opinion.

## Data Availability

The datasets generated and/or analyzed during this study are not publicly available as they include sensitive data of Medexo. This means that there are concerns about economic interests of other second opinion platforms in Germany, Austria and other countries. Due to this reason, authors wish do not share this confidential patient data.

## Abbreviations

CT: Computed Tomography Scan
HRQoL: Health-related quality of life
MRI: Magnetic Resonance Imaging
MSC-12: Mental Component Summary Score
PCS-12: Physical Component Summary Score
STROBE: Strengthening the Reporting of Observational Studies in Epidemiology
